# An Observational Study of Human Umbilical Cord Tissue Allografts for Paraspinal Muscle and Entheses Defects in the Thoracic and Lumbar Regions

**DOI:** 10.3390/biomedicines14051030

**Published:** 2026-04-30

**Authors:** Conrad Tamea, Jeff Buchalter, Jason Capra, Tracie Gilliland, Naomi Lambert, Alexis Lee, Tyler Barrett

**Affiliations:** 1 Orthopedic Associates of Tampa Bay, 4505 N Armenia Ave #101, Tampa, FL 33603, USA; 2 Professional Medical Consultants, 698 Brent Lane, Pensacola, FL 32503, USA; jbuchalter@professionalmedicalconsultants.com (J.B.);; 3 Advanced Medicine of the Ozarks, 104 Dyer Street, Mountain Home, AR 72653, USA; 4 Regenative Labs, 1700 W Main St., Pensacola, FL 32502, USA

**Keywords:** thoracic paraspinal muscles, lumbar paraspinal muscles, paraspinal muscle degeneration, paraspinal entheses, umbilical cord tissue, regenerative medicine

## Abstract

**Introduction:** With age and injury, the infiltration of fat in the paraspinal muscles can cause degeneration, disorganizing the structural integrity of the connective tissue and causing lower back pain (LBP). Human umbilical cord tissue allografts (UCTAs) have a collagen-rich matrix with various extracellular matrix (ECM) components that can replace damaged connective tissue. The objective of this paper is to analyze the preliminary findings from an observational repository on UCTAs for the supplementation of degenerated tissue in thoracic and lumbar paraspinal muscles refractory to standard conservative methods through patient-reported scales. **Materials and Methods:** A total of 117 patients from an observational repository were identified with paraspinal muscle degeneration. Patients received one to three applications of UCTAs; outcomes were tracked using the Numeric Pain Rating Scale (NPRS), the Western Ontario and McMaster University Arthritis Index (WOMAC), and the Quality-of-Life Scale (QOLS). **Results:** All groups showed positive improvement in the NPRS and WOMAC scales. Multi-application groups revealed statistically significant differences in the analyses. No adverse events or complications were reported. **Discussion:** Limitations included a lack of a control group, non-standardized application protocol, and the increase in recall and response bias due to using patient-reported measures. **Conclusions:** This pilot investigation presents the preliminary effectiveness necessary in hypothesis generation for continued research through randomized controlled trials to validate efficacy, establish optimal dosage protocols, and compare UCTAs to other conservative interventions.

## 1. Introduction

Lower back pain is prevalent across all age groups, and increasing evidence suggests that atrophy of the paraspinal muscles may serve as an underlying source of pain [1,2,3]. This association has gained growing attention in recent years, as the degeneration and functional decline of these muscles are increasingly recognized as essential contributors to symptom persistence [1,2,4]. These muscles attach to most of the spine, especially the thoracolumbar spine, and are fundamental elements of the spine and the whole body [2]. Significant spinal instability and reduced health can occur when paraspinal muscles and entheses are injured through trauma, surgery, fatty infiltration, or repeated facet denervation, subsequently causing postural issues and various low back pain disorders (LBPDs) [2,5]. LBPDs are complex, multifactorial conditions and are the number one cause of disability, affecting populations of all ages, income statuses, and regions [2,6]. However, older populations and patients with spine-related factors are at an increased risk of developing LBPDs [2]. While symptoms of back fatigue, stiffness, pain, and weakness may be attributed to non-specific lower back pain, these symptoms can also indicate paraspinal injury [7]. The presence of these symptoms may cause individuals to adopt sedentary lifestyles, further deteriorating their spine and overall quality of life.

On account of the similarity between low back pain and paraspinal injury symptoms, MRI imaging is recommended to evaluate a patient’s fat content within the paraspinal muscles to identify fatty degeneration/fatty infiltration and associated enthesopathies [8,9]. High signal intensity in MRI assessments indicates areas of high fatty infiltration, where adipose tissue grows in non-adipose tissues [10]. Fatty infiltration is related to the loss of muscle function, impairment of musculature integrity, and disruption of the extracellular matrix, which leads to muscle degeneration and atrophy [8,9,10]. Several classifications for the severity of paraspinal muscle degeneration have been proposed, most commonly the Goutallier classification, which is a five-stage grading scale (Grades 0–4) that utilizes MRI to assess fatty infiltration in muscle [11,12,13]. Grade 0 indicates normal muscle mass with no fatty infiltration; Grade 1 shows a few fatty streaks within the muscle; Grade 2 reflects less than 50% fat infiltration; Grade 3 represents approximately 50% fat infiltration; and Grade 4 denotes more than 50% fat infiltration [11,13]. While MRI imaging is preferred, ultrasound may also be utilized to identify muscular degeneration and degenerative enthesopathies of the thoracic and lumbar paraspinal space [14,15]. Upon confirmation of the diagnosis, paraspinal injury interventions can either be conservative or surgical, depending on the severity.

Currently, the primary standard of care approach for any musculoskeletal disorders is exercise-based physical therapy, including lumbar stabilization exercise programs (LSEPs), which is recommended to increase stability and maintain proper posture [16,17]. Other non-invasive modalities include high-frequency ultrasound wave therapy, NSAIDs, or low-level laser therapy [16]. However, most non-invasive approaches focus on the symptoms and not the fundamental problem from which the patient suffers. Considerations for surgical options regarding spinal muscle injuries are rare and are only offered when over half of the muscle area has been compromised with fat [18]. Surgical interventions are not recommended for muscle repair because of the complications that can arise postoperatively, specifically severe muscle hematoma, myositis ossificans, and compartmental syndrome [18,19]. Other invasive methods that do not focus on muscle repair, such as Restorative Neurostimulation (ReActv8), have shown durable results at 3 years but carry various complications related to device implantation, including infection, migration, and malposition [20,21,22]. The conventional management of paraspinal injuries is typically limited to conservative approaches, and the need for alternative methods that provide effective and long-term improvements is essential in standard patient care for paraspinal injuries.

The recurrence of symptoms following the conservative management of paraspinal injuries remains a significant concern for both clinicians and patients, prompting efforts to evaluate more effective and lasting approaches. One alternative non-invasive method to consider is umbilical cord tissue (UCT), which is an umbilical cord-derived connective tissue with extracellular components to supplement and replace homologous tissues around the body [23,24]. UCT primarily comprises glycosaminoglycans, proteoglycans, hyaluronic acid, various growth factors, and collagen types I, II, III, IV, V, VI, XII, and XIV with type I being the most abundant [23,25]. The utilized allograft adheres to FDA regulations for minimal manipulation, thereby preserving all of the tissue’s biochemical and biophysical attributes in the final allograft. The distinctive biological characteristics of perinatal tissues like UCT, including their anti-inflammatory, anti-fibrotic, anti-microbial, and immune-privileged nature, position them as ideal low-risk transplantable tissue, mainly owing to their diminished immune reactions [26]. The collagen fibers in the extracellular matrix of UCT mirror the extracellular matrix of cartilage, dermal tissues, and tendons [24]. The structural similarities of UCT to the entheses and fascia support its applicability in various musculoskeletal regions. Studies have shown promising results using UCTAs in over 180 homologous use sites, including the rotator cuff and the sacroiliac (SI) joint [27,28]. Both studies observed statistically significant results, showing improvements in joint function, mobility, quality of life, and pain alleviation [27,28]. The current literature lacks published data on the safety and efficacy of umbilical cord tissue for the management of paraspinal muscle injuries. The primary function, structural composition, and components in UCTAs are consistent with functional properties relevant to paraspinal muscle tissues. This observational case study aims to evaluate the preliminary results of umbilical cord tissue in patients with structural degeneration in the paraspinal muscles.

## 2. Materials and Methods

### 2.1. Study Design

**Figure 1:**
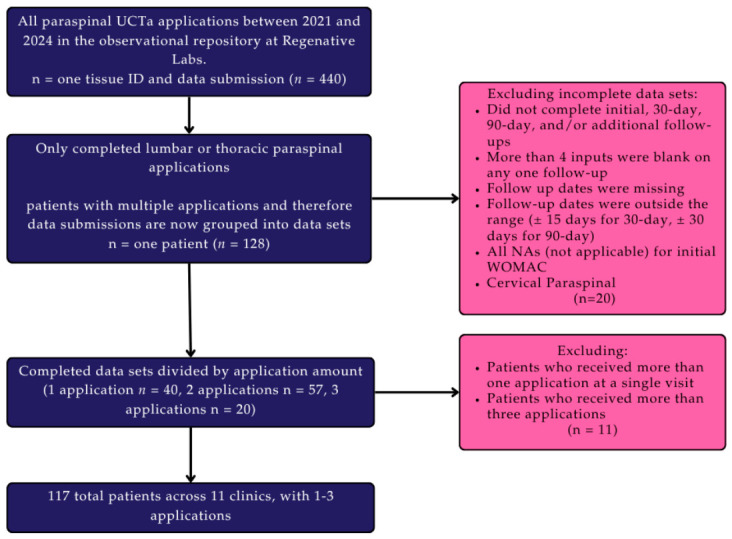
Flow chart of this study’s design.

The data from the observational repository at Regenative Labs were utilized in this study and have been maintained in compliance with the Declaration of Helsinki and approved by the Institutional Review Board of the Institute of Regenerative and Cellular Medicine (IRCM-2022-311) since January 2022. The study design of the repository and methods for UCTA processing are described in detail in other publications derived from the database [27,28]. The protocol covers observational data collection on all human perinatal tissue allografts offered by Regenative Labs between 2022 and 2025 (Regenative Labs, Pensacola, USA). The human umbilical cord tissue allografts were screened for communicable diseases and minimally manipulated according to the FDA’s 361 guidelines to maintain the original structure and function of the tissue for safe use in recipient patients. For participants to be eligible to receive flowable UCTAs, homologous use of the tissue must be justified—i.e., evidence of tissue degeneration, damaged or missing tissue, and exhausted and failed standard care as defined by Medicare for at least three months. As the data are observational, all application amounts and techniques were up to the discretion of each provider based on the severity and placement of the affected tissue. Patients submitted data at their initial application visit, 30 days post-allograft, and the primary endpoint was a 90-day follow-up to allow sufficient time for meaningful improvements after a single application, as clinically relevant changes are often observed within weeks and can be assessed by approximately 3 months [29]. Any reapplication of product extended the timeline by an additional 90 days. Reapplication was not required or standardized, only observed. Adverse events (AEs), any unfavorable medical occurrence related or unrelated to the research, were monitored through a 30 min post-application period, a 24–48 h patient monitoring period and at each subsequent follow up visit. Serious adverse events (SAEs), death or severe deterioration of health, were to be reported within 24 h of the event occurrence. AEs and SAEs were collected from signing the informed consent to the final follow-up visit. Informed consent was obtained from all patients before any applications were received. Multiple observers at multiple clinical sites were utilized to reduce observer bias. The inclusion criteria for patients in this post-observation period study included patients with lumbar or thoracic paraspinal degeneration, which was verified via MRI or ultrasound, depending on the provider, who received UCTA applications and had complete initial and follow-up data. Additional grading via Goutallier or other scales may have been used, but only imaging confirmation of a structural defect in the paraspinal muscles or entheses was required for the repository. Patients were excluded if they were lost to follow-up, if their data were out of the time range (±15 days for 30-day follow-up, ±30 days for 90-day, 120-day, 150-day, and 180-day follow-ups), if they reported all NAs (not applicable) at the initial visit, had multifaceted defects (i.e., paraspinal degeneration and degenerated discs), received multiple applications at a single visit, or received more than three applications. No exclusions were made based on gender, body mass index (BMI), or age. One hundred and seventeen patients from eleven different clinics met the criteria, and the final group sizes by number of applications for patients with one, two, and three applications are 46, 64, and 31, respectively. Figure 1 displays a flow chart of this study’s design.

### 2.2. Patient Procedures

**Figure 2:**
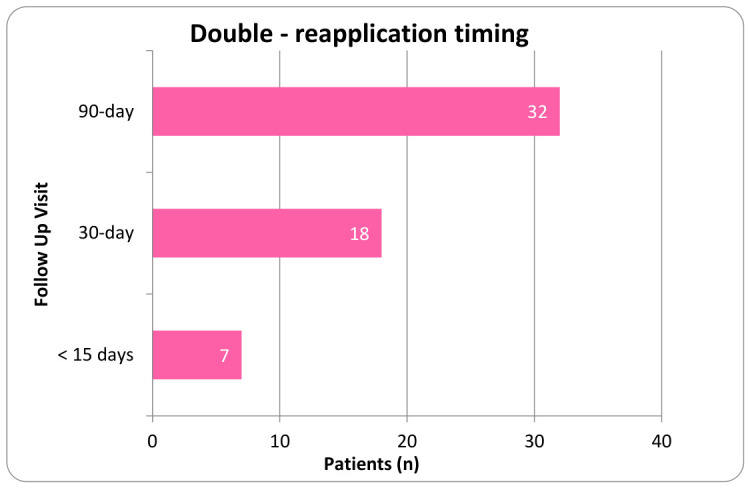
Number of patients who received their second application at each observed time point in the double application group.

**Figure 3:**
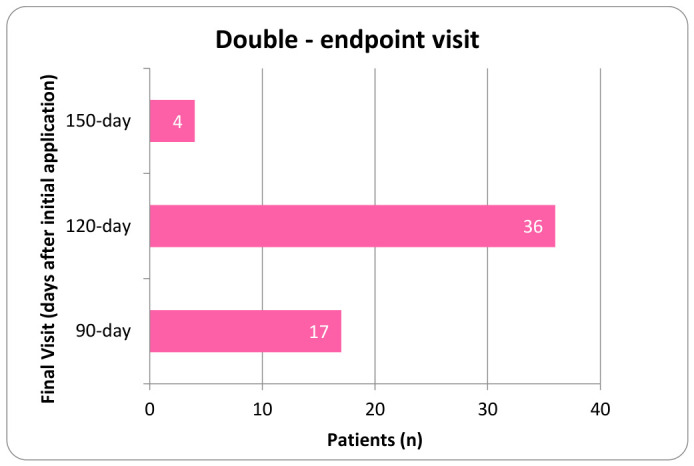
Number of patients who completed their observational period at each time point in the double application group.

**Figure 4:**
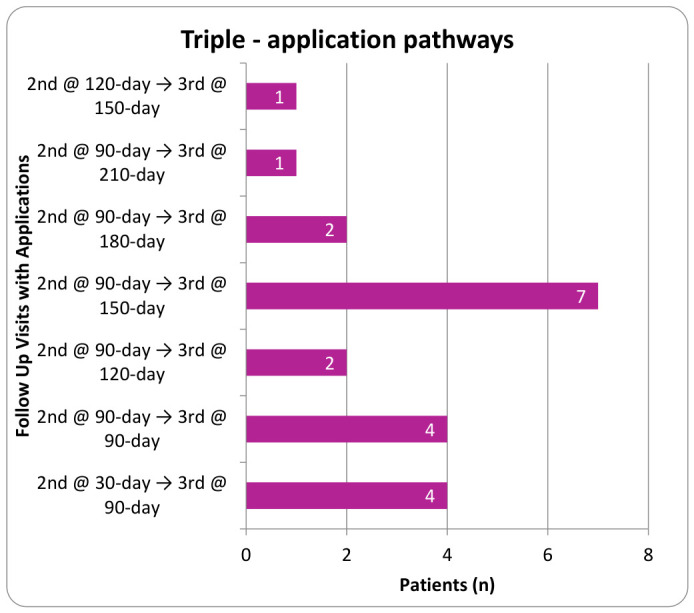
Number of patients who received their second and third application at the specified time points in the triple application group.

**Figure 5:**
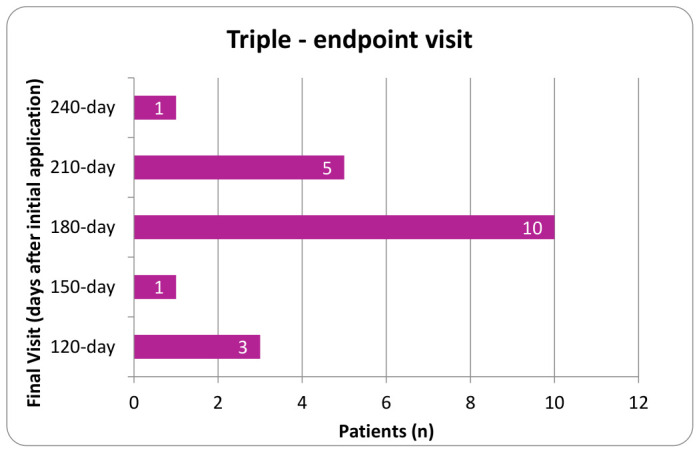
Number of patients who completed their observational period at each time point for the triple application group.

Procedures were performed using aseptic technique with ultrasound guidance. Patients were instructed to refrain from using anti-inflammatory drugs (NSAIDs) and corticosteroids for a minimum of two weeks before application and to avoid intra-articular corticosteroid injections within 90 days prior to the procedure. Before proceeding, physicians verified the correct patient identity, the planned procedure, the proper side and site, patient positioning, the availability of necessary implants, and any additional equipment or procedural requirements. Each clinical site maintained the right to perform its own technique, resulting in a slight variation in application protocols. The application site was prepared using a sterile technique. Anesthetic selection included either a topical coolant anesthetic or a local anesthetic such as Ropivacaine that was either added to the UCTA or administered intramuscularly prior to the UCTA. Using a 23 to 25-gauge 1.5-inch needle, two cc of 150 mg of cryopreserved umbilical cord tissue allograft, ProText™ (Regenative Labs, Pensacola, FL, USA) was applied into the targeted degeneration sites within the paraspinal muscles of the lumbar or thoracic region, as identified by ultrasound. Following application, the area was gently mobilized through its full range of motion, and a sterile dressing was placed. Post-procedure, patients were advised to apply ice or heat as needed for discomfort and to avoid strenuous activity for several days. For patients with multiple applications, the timing of subsequent applications was determined at the physician’s discretion within the designated evaluation period. In the double application cohort, the second application was applied anytime from the first week following their initial application to the 90-day visit. Patients in the third application group received their second and third applications between the first week following their initial visit to the 210-day follow-up. Figure 2, Figure 3, Figure 4 and Figure 5 display the timing distribution of reapplication for the double and triple application groups as well as the date of the final data set available.

### 2.3. Data Analysis

Patient-reported measures, including the Numeric Pain Rating Scale (NPRS), the Western Ontario and McMaster University Arthritis Index (WOMAC: Pain, Stiffness, Physical Function subscales, and Total), and the Quality-of-Life Scale (QOLS), assessed outcomes at initial and follow-up visits. Some patients reported missing NPRS values, and the difference in sample size for the NPRS testing is reflected in the outcomes table. No imputation was performed in this study. Descriptive statistics (mean, median, minimum, maximum, and standard deviation) were calculated for all scales across all intervals for each application group. The Shapiro–Wilk test was performed to identify statistically significant *p*-values, where *p* ≤ 0.05 indicated deviation from a normal distribution. The choice of parametric or non-parametric testing was determined by the normality assessment. Any comparison containing a time point that tested non-normally distributed received non-parametric testing. Within-group changes over time were assessed with pairwise comparisons. The Wilcoxon signed-rank test was used for non-normally distributed sets, and the paired *t*-test was used where all time points were normally distributed. Direct comparisons from initial to 90 days were also performed. For the double and triple application groups, an additional comparison from initial to the patient-specific endpoint (last available visit) was conducted to characterize the sustained treatment effect across the full follow-up period. For within-group paired comparisons, mean differences and 95% confidence intervals were calculated from the paired difference scores using the paired t-distribution (mean difference ± t_0.025_ × standard error of the difference). Cohen’s d was calculated for paired comparisons as the mean of the difference scores divided by the standard deviation of the difference scores. Effect sizes of |d| = 0.2, 0.5, and 0.8 correspond to small, medium, and large effects, respectively. Mean differences and effect sizes are reported for significant comparisons only; non-significant comparisons are reported by test statistic and *p*-value alone.

Between-group comparisons were conducted at fixed time points to ensure comparability across groups. Two complementary analyses were performed. Analysis A assessed outcome equivalence across all groups after a single application. Double and triple application groups were restricted to those who had received only one application at the time of their 90-day measurement (Double n = 32, Triple n = 16) and compared against the full single application group (n = 40). The Kruskal–Wallis test was used for omnibus comparison across all three groups at the 90-day visit.

Analysis B compared the single application group at 90 days (its natural endpoint) against the double and triple application groups at their respective final endpoints. This comparison was designed to evaluate the incremental benefit of additional applications relative to the outcome achievable with a single application. Kruskal–Wallis tests were used for omnibus comparisons, with Mann–Whitney U post-hoc tests applied where the omnibus test was significant (*p* < 0.05). Because the single application group had no follow-up data beyond 90 days, a fixed-time point three-way endpoint comparison was not possible. Double and triple application groups were therefore compared against single application groups at their respective last available visits rather than at a common time point. A direct comparison between double and triple application at their respective endpoints is also reported, though this comparison should be interpreted with caution, as the two groups had different mean follow-up durations (double approximately 120 days, triple approximately 180 days). For each significant post hoc comparison, the rank-biserial correlation (r) was reported as the effect size for the Mann–Whitney U test, calculated as r = 1 − 2U/(n_1_n_2_), where values of ±0.1, ±0.3, and ±0.5 correspond to small, medium, and large effects, respectively. The mean differences between groups and their 95% confidence intervals were estimated using 5000 bootstrap resamples with replacement (seed = 42, percentile method). Bonferroni correction was applied to post hoc tests within each outcome, using a multiplier of 3 to account for the three pairwise comparisons per outcome. Both raw and Bonferroni-adjusted *p*-values are reported in the appendix Table A1 and Table A2; the adjusted *p*-values are the primary criterion for determining the significance of post hoc comparisons.

The potential confounding effects of age, body mass index (BMI), and gender on outcome change scores were assessed across all three application groups. Change scores were defined as endpoint minus baseline, where endpoint was the 90-day visit for the single application group and the last available visit for the double and triple application groups, which is consistent with the primary endpoint definition used throughout. Spearman rank correlations were used to examine associations between age and BMI and continuous change scores given the non-normal distributions observed in several outcomes. Gender differences in change scores were evaluated using the Mann–Whitney U test.

Because BMI data were available for only a subset of patients (n = 19 of 40 single application, n = 19 of 57 double application, and n = 7 of 20 triple application), bootstrap confidence intervals (95%, 5000 resamples with replacement, seed = 42, percentile method) were computed for all Spearman correlations to provide more reliable interval estimates given the small subgroup sizes. Where bootstrap confidence intervals were not estimable—specifically for the triple application BMI subgroup (n = 7) due to insufficient variation—results are reported as point estimates only. Where bootstrap confidence intervals crossed zero despite a statistically significant *p*-value, findings were flagged as potentially unstable and interpreted with caution. A significance threshold of *p* < 0.05 was applied throughout. All statistical analyses were performed using RStudio (Version 2026.1.1.403, Posit Software, Boston, MA, USA) using the dplyr, tidyr, rstatix, ggplot2, and boot packages. The R analysis script used in this paper was drafted with assistance from Claude (Anthropic, San Francisco, CA, USA, claude.sonnet-4-6, 2026). All code was reviewed, verified, and executed by the authors, who take full responsibility for the analytical outputs.

## 3. Results

The patient cohort of 117 participants had 49% male participants, 50% female participants, and 1% unreported gender participants. The cohort was divided into three groups based on dosage frequency. Table 1 summarizes the patient characteristics.

**Figure 6:**
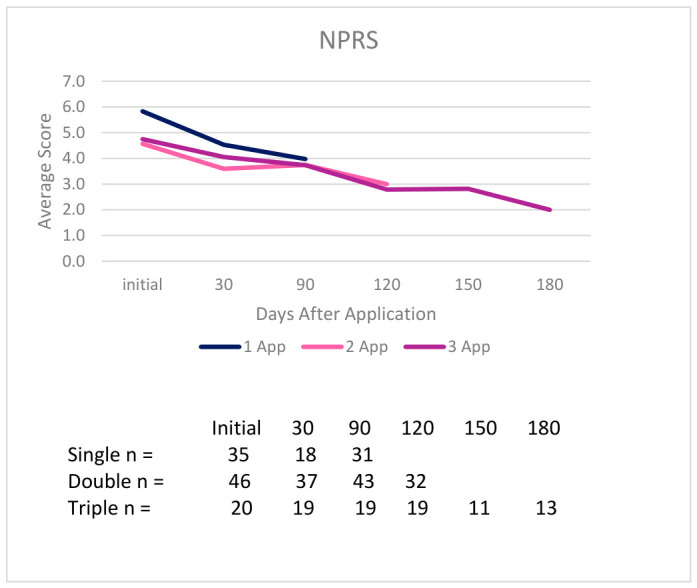
Average scores over time in the NPRS for each group with the amount of patients per time point in the legend chart.

**Figure 7:**
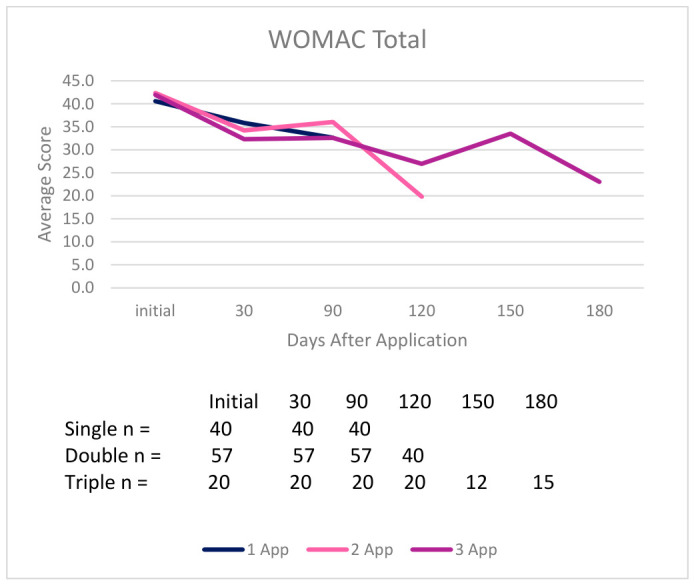
Average scores over time in the WOMAC total for each group with the amount of patients per time point in the legend chart.

**Figure 8:**
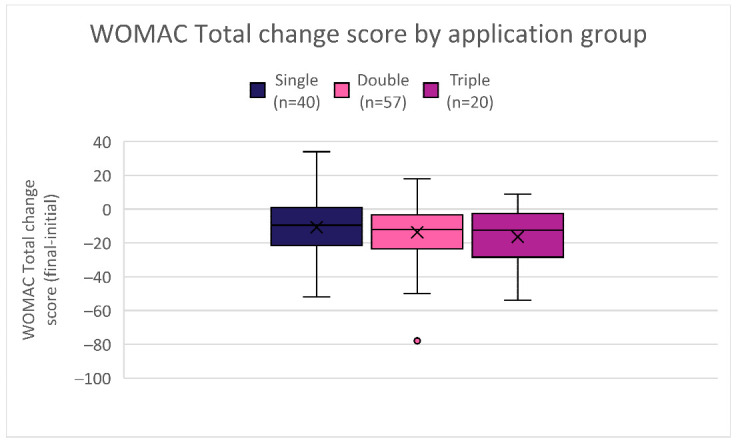
WOMAC Total change score distribution by application group.

It is important to note that a reduction in NPRS and WOMAC scores indicates improvement, and an increase in QOLS scores indicates improvement. In the single application group, 20 out of 29 patients who submitted an NPRS score at the initial and final visits reported improvement, 27 out of 40 patients reported improvement in total WOMAC scores, and 21 out of 40 patients reported improvement in QOLS scores. Twenty-two out of 46 patients in the double application cohort who had initial and final NPRS scores reported improvement, 46 out of 57 patients reported improvement in total WOMAC scores, and 32 out of 57 patients reported improvement in QOLS scores. The triple application group reported that 15 out of 18 patients experienced improvements in NPRS scores, 17 out of 20 patients reported improvements in total WOMAC scores, and 10 out of 20 patients reported improvements in QOLS scores. While fluctuations in the multi-application groups over time are typical due to rises in pain, indicating the need for a reapplication, reductions in overall score differences are still observed from the initial to final follow-up. Table 2 presents the average percent improvement in each scale from the initial to final visit across all dosage groups, and Figure 6, Figure 7 and Figure 8 display the average scores from initial to final visit in all dosage groups.

Mean scores for NPRS, WOMAC (Pain, Stiffness, Physical Function subscales, and Total), and QOLS across initial, 30-day, 90-day, and final visit scores demonstrate a positive trend of improvement in all application groups. Full descriptive statistics, including the mean, median, range, and normality are presented in Appendix A. Across all dosage groups, the NPRS and WOMAC scores decreased from the initial to the final visits. QOLS scores increased from baseline to final assessment in the multi-application groups, but in the single application group, a slight decrease was observed. A summary of the mean NPRS, WOMAC and QOLS scores from initial to endpoint with standard deviation in parenthesis is as follows:

- NPRS scores decreased as outlined below:
  ○ 6.54 (2.64) to 4.65 (2.73) (single application);
  ○ 4.41 (2.71) to 3.17 (2.29) (double application);
  ○ 4.75 (2.63) to 2.00 (1.75) (triple application).
- WOMAC Total scores decreased as outlined below:
  ○ 49.72 (21.14) to 38.98 (18.21) (single application)
  ○ 43.56 (17.48) to 29.84 (17.34) (double application)
  ○ 41.95 (20.62) to 25.55 (21.52) (triple application)
- QOLS scores increased as outlined below:
  ○ 75.70 (19.73) to 77.97 (15.68) (single application)
  ○ 82.11 (16.95) to 82.82 (16.69) (double application)
  ○ 77.90 (15.47) to 79.60 (20.26) (triple application)

Wilcoxon signed-rank tests and paired-t tests confirmed statistically significant improvements in the WOMAC (subscales and total) between initial, 30-day, 90-day, and endpoint intervals for all three application groups (Table 3). At the 30-day follow-up, the single application group demonstrated significant improvements from baseline in WOMAC Pain (W = 131.5, *p* = 0.008), Stiffness (W = 102.0, *p* = 0.020), Physical Function (W = 157.5, *p* = 0.017), and WOMAC Total (W = 149.5, *p* = 0.011). NPRS and QOLS did not change significantly at the 30-day follow-up, while the single application group demonstrated significant improvements from baseline in WOMAC Pain (W = 131.5, *p* = 0.008), Stiffness (W = 102.0, *p* = 0.020), Physical Function (W = 157.5, *p* = 0.017), and WOMAC Total (W = 149.5, *p* = 0.011). NPRS and QOLS did not change significantly at 30 days. By 90 days, significant improvements from baseline were maintained for all primary outcomes, as seen by the mean differences from baseline: WOMAC Total −10.75 points (95% CI −16.8 to −4.7, r = 0.609, n = 40); Pain −2.25 (95% CI −3.54 to −0.96, r = 0.590); Stiffness −1.15 (95% CI −1.83 to −0.47, r = 0.665); Physical Function −7.35 (95% CI −11.76 to −2.94, r = 0.583); and NPRS −2.55 (95% CI −3.54 to −1.56, r = 0.921, n = 29). All primary outcomes used the Wilcoxon signed-rank test; r denotes rank-biserial correlation. Consecutive 30-day to 90-day comparisons were non-significant for all outcomes, indicating that the primary application effect was established by 30 days and maintained through 90 days. QOLS showed a significant change from 30 days to 90 days (t = −2.790, *p* = 0.008, d = +0.45, n = 38) but was non-significant from baseline to 90 days (*p* = 0.322).

The double application group showed significant improvements from baseline at 30 days across all WOMAC outcomes and NPRS, as seen by the mean differences from baseline at the patient-level endpoint (last available visit): WOMAC Total −13.72 points (95% CI −18.25 to −9.19, r = 0.775, n = 57); Pain −2.53 (95% CI −3.60 to −1.46, r = 0.718); Stiffness −0.96 (95% CI −1.47 to −0.45, d = −0.50, paired *t*-test); Physical Function −10.23 (95% CI −13.47 to −6.99, r = 0.811); and NPRS −1.13 (95% CI −2.19 to −0.07, n = 38, Wilcoxon *p* = 0.052, borderline non-significant). WOMAC Total, Pain, Stiffness, and Physical Function all reached significance at endpoint (all *p* < 0.001). QOLS did not change significantly at any time point. WOMAC Total declined from a baseline mean of 43.6 to 29.8 at endpoint, representing a 31.7% improvement.

The triple application group demonstrated significant improvements from baseline at 30 days for all primary outcomes. At endpoint, the mean differences from baseline were WOMAC Total −16.40 points (95% CI −24.48 to −8.32, r = 0.867, n = 20); Pain −3.75 (95% CI −6.31 to −1.19, r = 0.904); Stiffness −1.45 (95% CI −2.99 to +0.09, r = 0.768); Physical Function −11.20 (95% CI −16.69 to −5.71, r = 0.936); and NPRS −2.56 (95% CI −3.84 to −1.28, r = 0.860, n = 18). All WOMAC outcomes and NPRS were significant at endpoint (all *p* ≤ 0.003); r denotes rank-biserial correlation (Wilcoxon signed-rank). The Stiffness CI marginally crossed zero, but the point estimate was clinically meaningful. QOLS was non-significant at all time points. WOMAC Total declined from a baseline mean of 42.0 to 25.6 at endpoint, representing a 39.1% improvement. The full results of this test including *p*-values, mean difference, CIs, and Cohen’s d or rank-biserial r depending on normality are presented in Table A2 of Appendix A.

Analysis A of the between-group comparisons evaluated the 90-day scores of patients across all groups who only had one application completed by the 90-day visit. When the double and triple application groups were restricted to patients who had received only one application at the time of their 90-day measurement (Double n = 32, Triple n = 15), no significant between-group differences were found on any outcome compared with the full single application group (n = 40): NPRS (H = 4.838, *p* = 0.089), Pain (H = 5.062, *p* = 0.080), Stiffness (H = 4.470, *p* = 0.107), Physical Function (H = 3.915, *p* = 0.141), WOMAC Total (H = 4.001, *p* = 0.135), and QOLS (H = 1.023, *p* = 0.600) (Table 4). This finding indicates that the three patient groups were achieving equivalent outcomes when at the same stage of care, supporting their baseline comparability and suggesting that subsequent differences may be attributable to additional applications rather than pre-existing group characteristics.

When the single application group at 90 days was compared against the double and triple application groups at their respective final endpoints, significant omnibus differences were found for NPRS (H = 14.142, *p* < 0.001), Pain (H = 9.954, *p* = 0.007), Stiffness (H = 8.558, *p* = 0.014), Physical Function (H = 8.946, *p* = 0.011), and WOMAC Total (H = 9.718, *p* = 0.008). QOLS was non-significant (H = 2.076, *p* = 0.354) (Table 5). Effect sizes and 95% CIs for all primary post hoc comparisons are reported below; Bonferroni-adjusted *p*-values apply a multiplier of 3 (three post hoc pairs per outcome) (Table 6).

Single versus double at endpoint: WOMAC Total MD = −9.13 points (95% CI −16.24 to −2.03, r = −0.32, *p* = 0.008, *p*_a_^dj^ = 0.024); Pain MD = −1.96 (95% CI −3.56 to −0.39, r = −0.30, *p* = 0.011, *p*_a_^dj^ = 0.033); Physical Function MD = −6.60 (95% CI −11.69 to −1.49, r = −0.31, *p* = 0.009, *p*_a_^dj^ = 0.028); NPRS MD = −1.48 (95% CI −2.62 to −0.29, r = −0.33, *p* = 0.015, *p*_a_^dj^ = 0.046). Stiffness was not significant after correction (MD = −0.57, 95% CI −1.18 to +0.04, r = −0.22, *p* = 0.065, *p*_a_^dj^ = 0.195).

Single versus triple at endpoint: WOMAC Total MD = −13.42 points (95% CI −23.93 to −2.82, r = −0.40, *p* = 0.013, *p*_a_^dj^ = 0.040); Pain MD = −3.30 (95% CI −5.50 to −1.12, r = −0.42, *p* = 0.008, *p*_a_^dj^ = 0.024); Stiffness MD = −0.75 (95% CI −2.20 to +1.20, r = −0.44, *p* = 0.006, *p*_a_^dj^ = 0.017); NPRS MD = −2.65 (95% CI −3.83 to −1.40, r = −0.60, *p* < 0.001, *p*_a_^dj^ = 0.001). Physical Function was borderline after correction (MD = −9.38, 95% CI −16.55 to −2.12, r = −0.37, *p* = 0.019, *p*_a_^dj^ = 0.058). Note that the Stiffness CI crossed zero despite a significant *p*-value, indicating limited precision.

Double versus triple at endpoint: No significant differences were found on any outcome after Bonferroni correction. NPRS showed the smallest *p*-value (MD = −1.17, 95% CI −2.17 to −0.12, r = −0.32, *p* = 0.045, *p*_a_^dj^ = 0.134) but did not survive correction. WOMAC Total MD = −4.29 (95% CI −14.34 to +6.02, r = −0.16, *p* = 0.293). All other outcomes were non-significant with CIs crossing zero.

Considerable individual variation in response was observed across all three groups (Table 7). In the single application group, WOMAC Total change scores at 90 days ranged from −52 (best response) to +34 (worst response) with a mean change of −10.75 and an outcome range of 86 points. The double application group showed a wider range at endpoint, from −78 to +18, with a mean change of −13.72 and a range of 96 points. The triple application group demonstrated the most consistent improvement at endpoint, ranging from −54 to +9 with a mean change of −16.40 and a range of 63 points. The narrowing of the worst response and range in the triple group suggests that additional applications may reduce the proportion of patients who experience no benefit or worsening, although the small sample size (n = 20) warrants caution in this interpretation.

No significant associations between age, BMI, or gender and outcome change scores were identified in the single application group, providing no evidence that baseline characteristics confounded the single-application outcomes (Table 8). Gender was non-significant for all outcomes across all three groups. In the double application group, higher BMI was significantly associated with smaller improvements in Stiffness at endpoint (r = −0.515, 95% bootstrap CI [−0.861, −0.004], *p* = 0.024, n = 19) and QOLS at endpoint (r = −0.703, 95% bootstrap CI [−0.872, −0.410], *p* < 0.001, n = 19). Both findings had bootstrap confidence intervals entirely below zero, indicating robustness despite the small BMI subgroup. No significant associations were found between BMI and WOMAC Total, Pain, Physical Function, or NPRS change scores in the double group, suggesting the BMI effect may be specific to stiffness and quality-of-life outcomes. Age was non-significant for all double application outcomes.

In the triple application group, older age was significantly associated with smaller improvement in QOLS at endpoint (r = −0.751, 95% bootstrap CI [−0.926, −0.409], *p* < 0.001, n = 20) with a robust confidence interval entirely below zero. Age was non-significant for all other outcomes in the triple group. BMI results for the triple group are reported as point estimates only (n = 7) and should not be interpreted given the insufficient sample size for this subgroup. Taken together, the confounder findings suggest that while age and BMI may influence specific quality-of-life and stiffness outcomes in multi-application patients, they are unlikely to be driving the primary between-group differences in WOMAC Total, Pain, and Physical Function. These primary outcomes were non-significantly associated with all covariates in all groups.

## 4. Discussion

The findings from this observational research study demonstrate the clinical potential of umbilical cord tissue (UCT) allografts to supplement paraspinal muscle and enthesis defects in the thoracic and lumbar spinal region. All patient groups reported lower average NPRS and WOMAC scores by the final visit compared to the initial visit as well as higher average QOLS scores at the final follow-up compared to the initial visit. Normality testing determined if the Wilcoxon signed-rank test or paired-t test was performed to identify statistically significant differences in all dosage groups at initial, 30-day, 90-day, and final visit intervals. Statistically significant differences were found in all three application groups, most notably in the WOMAC subscales and total, confirming the suspected benefits of UCTAs. Looking at the effect size for WOMAC Total outcomes between the baseline and the endpoint, all are considered large [30]. Still, r increases with each application: r = 0.609 in the single group, r = 0.775 in the double group, and r = 0.867 in the triple group.

Due to variable endpoints in the multi-application group, the last available visit was used to best compare the patient outcomes at the end of care in each group regardless of date. To compensate for the distribution of application times, we first analyzed all three groups together using only the patients who had received one application by the 90-day visit, n = 40 in the single group, n = 32 in the double group, and n = 15 in the triple group. Analysis A in Table 4 shows no significant differences between the groups in any scale, suggesting equivalent outcomes among patients with one application. Equivalent outcomes from a single application do not necessarily indicate equivalent response, as the single application had notably higher initial NPRS, pain, and stiffness scores than the multi-application groups. This brings to light the limitations of observational research, as the clinicians maintained full selection control and applied and reapplied based on their own evaluation of medical necessity for each patient, introducing selection bias. For this reason, initial scores cannot be used as an indicator for ideal dosage protocols; however, additional analysis of endpoint outcomes assists in hypothesis generation for dosing protocols. Analysis B in Table 5 comparing the last visit from all patients in each group reveals significant differences between the multi-application groups and the single application group. Post hoc pairwise comparisons with bootstrapping and Bonferroni’s correction confirm that the multi-application groups did significantly better by the end of care compared to the single application group in most scales except for QOLS with a moderate effect size ranging from r = −0.30 to r = −0.60 with the negative indicating lower scores, greater improvement, in the multi-application groups. The observed trends align with previous studies, where repeated exposure may reinforce and sustain outcomes in regenerative applications [31,32,33]. As this study was limited by non-standardized reapplication schedules, future studies focused on long-term benefits and controlled dosage applications would benefit the solidification of optimal dosage protocols and durability of the allografts in patients with treatment-resistant thoracic or lumbar paraspinal defects.

Beyond statistical significance, the outcome range analysis provided additional insight into possible dosage–response relationships. The larger outcome ranges observed in the single application group suggested greater variability in individual responsiveness, whereas higher dosage groups demonstrated narrower ranges, excluding one outlier in the double application group with extreme improvement, indicating a more consistent application response. As shown in Figure 8, changes in WOMAC Total score distributions for multi-application protocols not only improved outcomes but did so with greater consistency, as evidenced by the lower ranges. Not all subscales reflected this trend, but as preliminary data, the collective WOMAC Total range provides the best representation of overall patient outcomes. Multiple applications appeared to support a more sustained response, paralleling the progressive improvements observed across the higher-dosage groups in other statistical testing. When combined with the statistical findings, the outcome range analysis strengthens the hypothesis that multi-applications provide the greatest overall improvement within the patient population.

Spearman rank correlations with 95% bootstrap confidence intervals were used to examine the potential influence of age, BMI, and gender on outcome change scores across all three application groups. No significant associations were identified in the single application group, supporting the internal validity of the single-application findings. In the double application group, higher BMI was significantly associated with smaller improvements in stiffness (r = −0.515, 95% CI [−0.861, −0.004]) and quality of life as measured by QOLS (r = −0.703, 95% CI [−0.872, −0.410]), with both bootstrap confidence intervals lying entirely below zero, indicating these findings are robust to sampling variability. In the triple application group, older age was significantly associated with smaller improvement in QOLS (r = −0.751, 95% CI [−0.926, −0.409]). These findings suggest that BMI and age may modulate the response to UCTAs in specific domains, particularly quality of life and stiffness, in patients receiving multiple applications. Importantly, neither BMI nor age was significantly associated with changes in WOMAC Total, Pain, or Physical Function in any group, indicating that the primary between-group differences on these outcomes are unlikely to be explained by these demographic characteristics. Gender was the only factor that was not significant in any analysis; nonetheless, gender was analyzed given its frequent consideration in musculoskeletal research and its potential influence on functional recovery [34,35,36]. These confounder findings should be interpreted with caution given the small BMI subgroup sizes (n = 19 for single and double, n = 7 for triple), and replication in larger samples is warranted before drawing firm clinical conclusions about the moderating role of BMI and age on outcomes.

The encouraging outcomes from this pilot investigation should be interpreted within the context of several limitations. The design of this study was observational-based without a randomized control group, which limits direct comparisons with standard interventions and does not fully account for potential placebo effects. The follow-up duration was relatively short, preventing conclusions about long-term durability, sustained benefits, or late complications due to its focus on early responses. The patient procedural timing for multi-application was not standardized, resulting in variability in intervals of subsequent applications and final visit dates. The clinical outcome measures utilized were patient-reported, which are valuable for capturing subjective improvements in pain and function, but they may increase the possibility of response and recall bias. Because pain is subjective, the need for reapplication would have also been subjective to the evaluation of the provider, introducing selection bias despite evidence of structural defects. In addition, baseline and follow-up imaging, which would have strengthened the interpretation of structural changes, were not provided by the observers in this study. Future randomized controlled studies incorporating imaging and longer follow-ups are warranted to validate these preliminary findings.

While these considerations should be accounted for in interpreting the results, the analogous collagenous composition found in the paraspinal muscle and UCTAs strongly supports the biological plausibility of UCTAs when used in homologous locations. In paraspinal muscles, the primary component of the extracellular matrix (ECM) is type I collagen with other minor types including III, V, IX, and XI [37,38]. When these muscles are affected by injury, collagen III appears in the early wound-healing phase because of its loosely arranged fibrous network, providing elasticity and compliance [39,40]. As healing progresses, the cross-linking collagen I fibers, which provide mechanical strength and stability, replace collagen III fibers [39]. However, injured tissues rarely reach their maximum potential, regaining only 50–80% of the initial tensile strength [40]. ECM remodeling may become dysregulated, leading not to functional tissue restoration but to fibrotic repair characterized by excessive collagen deposition and abnormal cross-linking [39]. In the aging skeletal muscle, pathways associated with ECM remodeling are further impaired, resulting in an accumulation of densely cross-linked collagen type I fibers and increased tissue stiffness rather than improved structural integrity [39]. The increased collagen may reflect degenerative changes and fatty infiltration in paraspinal muscles, suggesting impaired adaptive ECM remodeling rather than insufficient collagen [41]. UTC allograft supplementation does not alter underlying ECM remodeling; instead, it provides structural support within compromised tissue. In UCT, the primary collagen found is type I with others including types II, III, IV, V, VI, XII, and XIV [25]. The collagen-rich matrix in UCT forms a network of thin collagen fibers that create a mesh-like scaffold [42]. Other ECM components in UCT include proteoglycan-rich components that provide ground substance, modulating viscoelastic properties, and hyaluronic acid, which is a glycosaminoglycan that contributes to water retention and tissue hydration [43]. Moreover, the structural similarities between the paraspinal muscles and UCT provide a framework for their functions in resisting unwanted movement in their respective locations [25,44]. The umbilical cord tissue, when applied intramuscularly and to the enthesis at the site of deterioration, replaces damaged tissue due to the similar structures and provides extra support against stressors to the strained connective tissues.

While the lack of comparison groups is present in this study’s design, the preliminary results observed that UCT allografts may provide more consistent outcomes in patients than other alternative interventions. The widely known autologous regenerative medicine, platelet-rich plasma (PRP) therapy, has been most commonly observed in thoracic and lumbar discs, epidural space, and facet joints with emerging literature revealing applications in paraspinal muscles [45,46,47,48,49,50]. Case reports in small clinical series have described PRP injections into paraspinal muscles with improvements in pain and function and no serious complications [49,50,51]. Because PRP is not widely studied for paraspinal uses, the current literature stresses the imperative for standardized protocols in PRP therapy, as multiple studies have documented inconsistent outcomes in different areas of application [52,53]. These variations might arise from the requirement for specific platelet concentrations, underscoring the influence of individual patient health on application efficacy [52,54]. Because PRP is prepared from the patient’s own blood, variability in baseline platelet counts can markedly affect the final product and, consequently, therapeutic response [55]. Because of the collection and processing methods used for human UCTAs, the final product is not dependent on patient health. Human UCTAs are rigorously screened using processing protocols that provide a high level of confidence that tissue quality remains consistent across products. These allografts have shown consistency across various homologous use sites, offering pain relief and increased functionality by replacing damaged tissue, as reported in previous publications from the same repository [27,28]. As an emerging conservative approach, UCTA applications could be a standardized option before surgical intervention to preserve muscle integrity. Continued research ought to feature study designs including comparative analyses and randomized clinical trials, and the safety, feasibility, and efficacy of these initial findings might be confirmed. Such confirmation would provide definitive evidence for the clinical application of UCTA supplementation in addressing paraspinal muscle defects in the thoracic and lumbar spine that are refractory to standard practices.

## 5. Conclusions

A total of 117 participants were evaluated after receiving either a single, double, or triple application of umbilical cord allografts. The results revealed no adverse events or complications with improvements in pain, stiffness, functionality, and quality of life over a 90–180-day period with statistical significance in multiple tests. Although the findings are promising, the use of patient-reported outcomes, the lack of a control group, variations in the timing of subsequent UCTA applications, and the short observation period preclude conclusive proof of the effectiveness of UCTA application. These preliminary findings generate the hypothesis that UCTAs for paraspinal muscle defects in the thoracic and lumbar regions are safe, feasible, and clinically applicable before surgical intervention is considered. Further investigation is warranted to determine the specific role of this regenerative approach when clinically managing the degeneration of the thoracic and lumbar paraspinal muscles.

## Figures and Tables

**Table 1:** Patient gender, mean BMI, by application amount and their mean age and BMI for each application category.

Characteristic	Single (n = 40)	Double (n = 57)	Triple (n = 20)
Gender (M/F)	20/20	27/29 (1 unknown)	10/10
Age mean (SD)	68.2 (9.3)	71.1 (9.6)	70.9 (5.6)
Age range	36–82	36–91	56–81
BMI mean (SD)	27.8 (5.3), n = 19	30.0 (3.1), n = 19	29.5 (7.3), n = 7

**Table 2:** Mean percentage change in each scale by number of applications from the initial to the last data set reported. For NPRS and WOMAC subscale, a decrease in scores equates to improvement, while the inverse is true for QOLS.

Scale	1 App	2 App	3 App
NPRS	−29.00%	−28.24%	−57.89%
WOMAC Total	−21.62%	−31.49%	−39.09%
Pain	−21.03%	−28.02%	−42.13%
Stiffness	−23.23%	−23.01%	−32.22%
Functionality	−21.57%	−33.70%	−39.23%
QOLS	+3.01%	+0.88%	+2.18%

**Table 3:** Significance of pairwise time point comparisons for all scales in each group, normality testing determined the type of testing used.

Outcome	Initial → 30 d	Initial → 90 d	Initial → End	30 d → 90 d
Single application (n = 40)				
NPRS	NS	↓ Sig.	—	NS
Pain	↓ Sig.	↓ Sig.	—	NS
Stiffness	↓ Sig.	↓ Sig.	—	NS
Physical Function	↓ Sig.	↓ Sig.	—	NS
WOMAC Total	↓ Sig.	↓ Sig.	—	NS
QOLS	NS *	NS *	—	↑ Sig. *
Double application (n = 57)				
NPRS	↓ Sig.	NS	NS	NS
Pain	↓ Sig.	↓ Sig. *	↓ Sig.	NS *
Stiffness	↓ Sig.	NS	↓ Sig. *	NS
Physical Function	↓ Sig. *	↓ Sig. *	↓ Sig.	NS *
WOMAC Total	↓ Sig. *	↓ Sig. *	↓ Sig.	NS *
QOLS	NS	NS *	NS	NS
Triple application (n = 20)				
NPRS	↓ Sig.	NS	↓ Sig.	NS
Pain	↓ Sig.	↓ Sig.	↓ Sig.	NS
Stiffness	↓ Sig.	↓ Sig. *	↓ Sig.	NS
Physical Function	↓ Sig.	↓ Sig.	↓ Sig.	NS
WOMAC Total	↓ Sig.	↓ Sig.	↓ Sig.	NS
QOLS	NS *	NS *	NS *	NS *

↓ Sig. = statistically significant decrease (*p* < 0.05; improvement for Pain, Stiffness, Physical Function, WOMAC Total, NPRS). ↑ Sig. = statistically significant increase (*p* < 0.05; improvement for QOLS). NS = not significant. — = comparison not applicable (single application group has no endpoint beyond 90 days). See Appendix A for *p*-values, effect size, and CIs. Wilcoxon signed-rank test used where any time point was non-normally distributed (Shapiro–Wilk *p* < 0.05); paired *t*-test used where all time points were normally distributed. End = 90 days for single application; last available visit for double and triple application groups. * = Paired *t*-test used.

**Table 4:** Kruskal–Wallis comparison of 90-day data restricted to patients with one application at 90 days.

Outcome	Single Mean	Double-1app Mean	Triple-1app Mean	KW *p*	Result
NPRS	4.65 (n = 40)	3.68 (n = 32)	3.33 (n = 15)	*p* = 0.089	NS
Pain	8.45 (n = 40)	6.82 (n = 32)	5.62 (n = 15)	*p* = 0.080	NS
Stiffness	3.80 (n = 40)	3.51 (n = 32)	2.88 (n = 15)	*p* = 0.107	NS
Physical Function	26.72 (n = 40)	23.92 (n = 32)	18.75 (n = 15)	*p* = 0.141	NS
WOMAC Total	38.98 (n = 40)	34.26 (n = 32)	27.25 (n = 15)	*p* = 0.135	NS
QOLS	77.97 (n = 40)	81.90 (n = 32)	81.94 (n = 15)	*p* = 0.600	NS

NS = Not Significant. No significant between-group differences when all patients had received only one application. Confirms outcome equivalence at the single-application stage.

**Table 5:** Outcome ranges for WOMAC outcome domains across the single application group.

Outcome	Single (90 d)	Double (Endpoint)	Triple (Endpoint)	KW *p*
NPRS	4.65 (n = 31)	3.17 (n = 42)	2.00 (n = 18)	***p* ≤ 0.001**
Pain	8.45	6.49	5.15	***p* = 0.007**
Stiffness	3.80	3.23	3.05	***p* = 0.014**
Phys. Func.	26.72	20.12	17.35	***p* = 0.011**
WOMAC Total	38.98	29.84	25.55	***p* = 0.008**
QOLS	77.97	82.82	79.60	*p* = 0.354

Values are group means. Kruskal–Wallis omnibus test. Significant *p*—values are bold.

**Table 6:** Post hoc pairwise comparisons with mean differences, 95% bootstrap CIs, rank-biserial r, and Bonferroni-adjusted *p*-values.

Outcome	MD	95% Bootstrap CI	r	*p* (raw)	*p* (adj)
**Single (90 d) vs. Double (endpoint)—Bonferroni multiplier = 3**					
NPRS	−1.48	[−2.62, −0.29]	−0.33	***p* = 0.015**	***p* = 0.046**
Pain	−1.96	[−3.56, −0.39]	−0.30	***p* = 0.011**	***p* = 0.033**
Stiffness	−0.57	[−1.18, +0.04]	−0.22	*p* = 0.065	*p* = 0.195
Phys. Function	−6.60	[−11.69, −1.49]	−0.31	***p* = 0.009**	***p* = 0.028**
WOMAC Total	−9.13	[−16.24, −2.03]	−0.32	***p* = 0.008**	***p* = 0.024**
QOLS	—	—	—	*p* = —	*p* = —
**Single (90 d) vs. Triple (endpoint)—Bonferroni multiplier = 3**					
NPRS	−2.65	[−3.83, −1.40]	−0.60	***p*** **= <0.001**	***p*** **= 0.001**
Pain	−3.30	[−5.50, −1.12]	−0.42	***p*** **= 0.008**	***p*** **= 0.024**
Stiffness	−0.75	[−2.20, +1.20]	−0.44	***p*** **= 0.006**	***p*** **= 0.017**
Phys. Function	−9.38	[−16.55, −2.12]	−0.37	***p*** **= 0.019**	*p* = 0.058
WOMAC Total	−13.42	[−23.93, −2.82]	−0.40	***p*** **= 0.013**	***p*** **= 0.040**
QOLS	—	—	—	*p* = —	*p* = —
**Double (endpoint) vs. Triple (endpoint)—Bonferroni multiplier = 3**					
NPRS	−1.17	[−2.17, −0.12]	−0.32	***p* = 0.045**	*p* = 0.134
Pain	−1.34	[−3.39, +0.75]	−0.18	*p* = 0.236	*p* = 0.709
Stiffness	−0.18	[−1.66, +1.76]	−0.24	*p* = 0.105	*p* = 0.315
Phys. Function	−2.77	[−9.46, +4.00]	−0.14	*p* = 0.367	*p* = 1.000
WOMAC Total	−4.29	[−14.34, +6.02]	−0.16	*p* = 0.293	*p* = 0.879
QOLS	—	—	—	*p* = —	*p* = —

MD = mean difference (group B minus group A). 95% CI from 5000 bootstrap resamples. r = rank-biserial correlation (effect size for Mann–Whitney U). *p* (adj) = Bonferroni-adjusted *p*-value (×3 per outcome). Negative MD and r indicate lower scores in the second group. — = not tested (QOLS omnibus NS). Significant *p*—values are bold.

**Table 7:** Outcome ranges for WOMAC outcome domains across all groups.

Scale	Group	Best Response (Max Δ)	Worst Response (Min Δ)	Mean Change (Δ)	Outcome Range (Δ)
WOMAC Total	Single (n = 40) (90-day)	−52	34	−10.75	86
Pain		−12	5	−2.25	17
Stiffness		−4	6	−1.15	10
Functionality		−39	25	−7.35	64
WOMAC Total	Double (n = 57) (Last visit)	−78	18	−13.72	96
Pain		−15	9	−2.53	24
Stiffness		−5	4	−0.96	9
Functionality		−59	12	−10.23	71
WOMAC Total	Triple (n = 20) (Last visit)	−54	9	−16.4	63
Pain		−23	2	−3.75	25
Stiffness		−6	11	−1.45	17
Functionality		−38	3	−11.2	41

Δ = change from baseline to endpoint. Single application endpoint = 90-day visit. Double and triple application endpoint = each patient’s last available visit. Negative values indicate improvement. Best Response = largest improvement (most negative Δ); Worst Response = largest worsening (most positive Δ).

**Table 8:** Spearman rank correlations between change scores and age/BMI across all three groups with 95% bootstrap confidence intervals (5000 resamples). Bootstrap CIs applied given small BMI subgroup sizes. Gender was non-significant for all outcomes in all groups and is not shown.

Group	Outcome	Covariate	r	*p*-Value	95% Bootstrap CI	Result
**Significant findings**						
Double	Stiffness change (endpoint)	BMI	−0.515	***p* = 0.024**	[−0.861, −0.004]	Yes *
Double	QOLS change (endpoint)	BMI	−0.703	***p* = <0.001**	[−0.872, −0.410]	Yes *
Triple	QOLS change (endpoint)	Age	−0.751	***p* = <0.001**	[−0.926, −0.409]	Yes *
**Non-significant—all groups (selected findings** **shown)**						
Single	All outcomes (90-day)	BMI, Age, Gender	—	*p* = All NS	—	NS
Double	Physical Function change	BMI	−0.016	*p* = 0.947	[−0.595, +0.511]	NS
Double	WOMAC Total change	BMI	−0.135	*p* = 0.582	[−0.685, +0.396]	NS
Triple	Stiffness change	Age	+0.398	*p* = 0.082	[−0.072, +0.737]	NS
Triple	Physical Function change	Age	+0.265	*p* = 0.259	[−0.209, +0.646]	NS

Bootstrap CIs based on 5000 resamples (seed = 42). All groups use endpoint change scores (Single = 90-day; Double/Triple = last available visit). Single: BMI n = 19, Age n = 29–40. Double: BMI n = 19, Age n = 46–57. Triple: BMI n = 7 (CIs not estimable), Age n = 20. Gender non-significant in all groups. The previously reported Triple Physical Function/Age finding (r = +0.452, *p* = 0.045 at 90-day) was not replicated at endpoint (r = +0.265, *p* = 0.259). * = significant finding. NS = not significant. — = comparison not applicable (single application group has no endpoint beyond 90 days). Significant *p*—values are bold.

## Data Availability

The original contributions presented in the study are included in the article, further inquiries can be directed to the corresponding author.

## Abbreviations

The following abbreviations are used in this manuscript:

UCT	Umbilical cord tissue
UCTA	Umbilical cord tissue allograft
WOMAC	Western Ontario and McMaster University Arthritis Index
NPRS	Numeric Pain Rating Scale
QOLS	Quality-of-Life Scale

## Appendix A

**Table A1:** Descriptive statistics for initial, 30-day, 90-day, and endpoint time intervals across all application groups with Shapiro–Wilk normality testing (*p* < 0.05).

Outcome	Time Point	n	Mean (SD)	Median	Range	SW *p*	Normal?
**Single Application (n = 40)**							
NPRS	Initial	35	6.54 (2.64)	7	2–10	0.002	No
NPRS	30-Day	18	5.56 (2.50)	6	1–9	0.008	No
NPRS	90-Day	31	4.65 (2.73)	5	0–10	0.402	Yes
Pain	Initial	40	10.70 (4.75)	11.5	1–19	0.004	No
Pain	30-Day	40	9.07 (3.88)	9.5	0–19	0.727	Yes
Pain	90-Day	40	8.45 (4.05)	8	0–17	0.393	Yes
Stiffness	Initial	40	4.95 (2.15)	6	0–8	0.001	No
Stiffness	30-Day	40	4.25 (1.75)	4	0–8	0.056	Yes
Stiffness	90-Day	40	3.80 (1.32)	4	1–6	0.025	No
Physical Function	Initial	40	34.08 (15.06)	36	2–57	0.008	No
Physical Function	30-Day	40	29.52 (12.78)	30.5	3–57	0.801	Yes
Physical Function	90-Day	40	26.72 (13.28)	26.5	0–54	0.821	Yes
WOMAC Total	Initial	40	49.72 (21.14)	51.5	4–83	0.005	No
WOMAC Total	30-Day	40	42.85 (17.60)	45	4–83	0.634	Yes
WOMAC Total	90-Day	40	38.98 (18.21)	37.5	2–75	0.775	Yes
QOLS	Initial	40	75.70 (19.73)	78.5	31–109	0.231	Yes
QOLS	30-Day	38	71.34 (16.89)	70.5	40–107	0.696	Yes
QOLS	90-Day	40	77.97 (15.68)	82	42–109	0.368	Yes
**Double Application (n = 57)**							
NPRS	Initial	46	4.41 (2.71)	5	0–10	0.007	No
NPRS	30-Day	37	3.35 (2.03)	4	1–8	0.002	No
NPRS	90-Day	43	3.81 (2.56)	3	0–10	0.004	No
NPRS	Endpoint	42	3.17 (2.29)	2	0–8	<0.001	No
Pain	Initial	57	9.02 (3.90)	9	1–17	0.108	Yes
Pain	30-Day	57	7.33 (3.82)	8	0–16	0.188	Yes
Pain	90-Day	57	7.26 (4.02)	7	0–17	0.099	Yes
Pain	Endpoint	57	6.49 (4.02)	6	0–16	0.029	No
Stiffness	Initial	57	4.19 (2.14)	5	0–8	0.006	No
Stiffness	30-Day	57	3.39 (1.94)	4	0–8	0.011	No
Stiffness	90-Day	57	3.74 (1.93)	4	0–8	0.056	Yes
Stiffness	Endpoint	57	3.23 (1.82)	3	0–7	0.015	No
Physical Function	Initial	57	30.35 (12.28)	31	4–63	0.445	Yes
Physical Function	30-Day	57	23.56 (11.52)	24	3–52	0.411	Yes
Physical Function	90-Day	57	24.88 (13.48)	26	2–59	0.070	Yes
Physical Function	Endpoint	57	20.12 (12.12)	18	2–54	0.046	No
WOMAC Total	Initial	57	43.56 (17.48)	45	6–83	0.280	Yes
WOMAC Total	30-Day	57	34.28 (16.30)	35	4–75	0.553	Yes
WOMAC Total	90-Day	57	35.88 (18.57)	36	6–83	0.085	Yes
WOMAC Total	Endpoint	57	29.84 (17.34)	26	3–77	0.054	Yes
QOLS	Initial	57	82.11 (16.95)	82	38–110	0.065	Yes
QOLS	30-Day	57	83.46 (22.39)	82	39–174	<0.001	No
QOLS	90-Day	57	81.14 (16.70)	83	42–112	0.216	Yes
QOLS	Endpoint	57	82.82 (16.69)	83	42–112	0.131	Yes
**Triple Application (n = 20)**							
NPRS	Initial	20	4.75 (2.63)	5	1–10	0.309	Yes
NPRS	30-Day	19	4.05 (2.48)	4	1–10	0.113	Yes
NPRS	90-Day	19	3.74 (3.00)	4	1–9	0.002	No
NPRS	Endpoint	18	2.00 (1.75)	1	0–7	<0.001	No
Pain	Initial	20	8.90 (6.05)	9	0–27	0.036	No
Pain	30-Day	20	6.00 (4.23)	6	0–14	0.306	Yes
Pain	90-Day	20	6.70 (4.57)	7	0–14	0.275	Yes
Pain	Endpoint	20	5.15 (4.31)	5.5	0–13	0.079	Yes
Stiffness	Initial	20	4.50 (1.67)	5	1–7	0.090	Yes
Stiffness	30-Day	20	3.35 (1.87)	4	0–6	0.015	No
Stiffness	90-Day	20	3.25 (1.97)	3	0–7	0.232	Yes
Stiffness	Endpoint	20	3.05 (3.98)	2	0–18	<0.001	No
Physical Function	Initial	20	28.55 (14.70)	31	3–48	0.042	No
Physical Function	30-Day	20	22.95 (12.86)	26.5	2–44	0.273	Yes
Physical Function	90-Day	20	22.65 (16.17)	25.5	0–45	0.036	No
Physical Function	Endpoint	20	17.35 (14.20)	16.5	0–43	0.100	Yes
WOMAC Total	Initial	20	41.95 (20.62)	46	6–66	0.021	No
WOMAC Total	30-Day	20	32.30 (18.38)	36	2–63	0.312	Yes
WOMAC Total	90-Day	20	32.60 (22.35)	35	0–65	0.067	Yes
WOMAC Total	Endpoint	20	25.55 (21.52)	24	0–72	0.153	Yes
QOLS	Initial	20	77.90 (15.47)	73	57–110	0.105	Yes
QOLS	30-Day	20	76.80 (14.71)	73.5	47–107	0.491	Yes
QOLS	90-Day	20	78.35 (19.63)	75.5	40–107	0.328	Yes
QOLS	Endpoint	20	79.60 (20.26)	77.5	45–106	0.057	Yes

SW = Shapiro–Wilk *p*-value. Endpoint row shown for double and triple groups only (last available visit per patient). Single application uses 90-day as its natural endpoint.

**Table A2:** All pairwise time point comparisons with mean differences (MD), 95% CIs, and effect sizes (Cohen’s d for paired *t*-test; rank-biserial r for Wilcoxon). MD and CI shown only for significant comparisons. Endpoint = last available visit for double and triple application.

Outcome	Comparison	Test	Statistic	*p*	MD	95% CI	d/r
**Single Application—All Comparisons**							
NPRS	Initial → 30-Day	Wilcoxon	W = 19.0	*p* = 0.208	—	—	—
NPRS	30-Day → 90-Day	Wilcoxon	W = 19.0	*p* = 0.112	—	—	—
NPRS	Initial → 90-Day	Wilcoxon	W = 10.0	***p ≤* 0.001**	−2.55	[−3.54, −1.56]	r = 0.921
Pain	Initial → 30-Day	Wilcoxon	W = 131.5	***p* = 0.008**	−1.62	[−2.75, −0.50]	r = 0.531
Pain	30-Day → 90-Day	Wilcoxon	W = 172.0	*p* = 0.478	—	—	—
Pain	Initial → 90-Day	Wilcoxon	W = 129.0	***p* = 0.002**	−2.25	[−3.54, −0.96]	r = 0.590
Stiffness	Initial → 30-Day	Wilcoxon	W = 102.0	***p* = 0.020**	−0.70	[−1.42, +0.02]	r = 0.498
Stiffness	30-Day → 90-Day	Wilcoxon	W = 164.0	*p* = 0.091	—	—	—
Stiffness	Initial → 90-Day	Wilcoxon	W = 83.0	***p* = 0.001**	−1.15	[−1.83, −0.47]	r = 0.665
Physical Function	Initial → 30-Day	Wilcoxon	W = 157.5	***p* = 0.017**	−4.55	[−8.25, −0.85]	r = 0.471
Physical Function	30-Day → 90-Day	Wilcoxon	W = 226.0	*p* = 0.144	—	—	—
Physical Function	Initial → 90-Day	Wilcoxon	W = 154.5	***p* = 0.002**	−7.35	[−11.76, −2.94]	r = 0.583
WOMAC Total	Initial → 30-Day	Wilcoxon	W = 149.5	***p* = 0.011**	−6.88	[−12.07, −1.68]	r = 0.497
WOMAC Total	30-Day → 90-Day	Wilcoxon	W = 230.5	*p* = 0.166	—	—	—
WOMAC Total	Initial → 90-Day	Wilcoxon	W = 152.5	***p* = <0.001**	−10.75	[−16.80, −4.70]	r = 0.609
QOLS	Initial → 30-Day	Paired *t*-test	t = 1.822	*p* = 0.077	—	—	—
QOLS	30-Day → 90-Day	Paired *t*-test	t = −2.790	***p* = 0.008**	+6.58	[+1.80, +11.36]	d = +0.45
QOLS	Initial → 90-Day	Paired *t*-test	t = −1.002	*p* = 0.322	—	—	—
**Double Application—All Comparisons**							
NPRS	Initial → 30-Day	Wilcoxon	W = 0.0	***p* = <0.001**	−0.89	[−1.33, −0.45]	r = 1.000
NPRS	30-Day → 90-Day	Wilcoxon	W = 171.5	*p* = 0.672	—	—	—
NPRS	Initial → 90-Day	Wilcoxon	W = 269.5	*p* = 0.843	—	—	—
NPRS	Initial → Endpoint	Wilcoxon	W = 161.0	*p* = 0.052	—	—	—
Pain	Initial → 30-Day	Paired *t*-test	t = 4.140	***p* = <0.001**	−1.68	[−2.50, −0.87]	d = −0.55
Pain	30-Day → 90-Day	Paired *t*-test	t = 0.160	*p* = 0.873	—	—	—
Pain	Initial → 90-Day	Paired *t*-test	t = 3.336	***p* = 0.002**	−1.75	[−2.81, −0.70]	d = −0.44
Pain	Initial → Endpoint	Wilcoxon	W = 180.0	***p* = <0.001**	−2.53	[−3.59, −1.46]	r = 0.718
Stiffness	Initial → 30-Day	Wilcoxon	W = 124.0	***p* = <0.001**	−0.81	[−1.21, −0.40]	r = 0.628
Stiffness	30-Day → 90-Day	Wilcoxon	W = 286.0	*p* = 0.141	—	—	—
Stiffness	Initial → 90-Day	Wilcoxon	W = 317.0	*p* = 0.088	—	—	—
Stiffness	Initial → Endpoint	Paired *t*-test	t = 3.781	***p* = <0.001**	−0.96	[−1.48, −0.45]	d = −0.50
Physical Function	Initial → 30-Day	Paired *t*-test	t = 5.698	***p* = <0.001**	−6.79	[−9.18, −4.40]	d = −0.75
Physical Function	30-Day → 90-Day	Paired *t*-test	t = −1.041	*p* = 0.303	—	—	—
Physical Function	Initial → 90-Day	Paired *t*-test	t = 3.357	***p* = 0.001**	−5.47	[−8.74, −2.21]	d = −0.44
Physical Function	Initial → Endpoint	Wilcoxon	W = 140.5	***p* = <0.001**	−10.23	[−13.47, −6.99]	r = 0.811
WOMAC Total	Initial → 30-Day	Paired *t*-test	t = 5.728	***p* = <0.001**	−9.28	[−12.53, −6.03]	d = −0.76
WOMAC Total	30-Day → 90-Day	Paired *t*-test	t = −0.898	*p* = 0.373	—	—	—
WOMAC Total	Initial → 90-Day	Paired *t*-test	t = 3.417	***p* = 0.001**	−7.68	[−12.19, −3.18]	d = −0.45
WOMAC Total	Initial → Endpoint	Wilcoxon	W = 186.0	***p* = <0.001**	−13.72	[−18.25, −9.19]	r = 0.775
QOLS	Initial → 30-Day	Wilcoxon	W = 258.5	*p* = 0.504	—	—	—
QOLS	30-Day → 90-Day	Wilcoxon	W = 434.5	*p* = 0.642	—	—	—
QOLS	Initial → 90-Day	Paired *t*-test	t = 0.590	*p* = 0.558	—	—	—
QOLS	Initial → Endpoint	Wilcoxon	W = 463.5	*p* = 0.093	—	—	—
**Triple Application—All Comparisons**							
NPRS	Initial → 30-Day	Wilcoxon	W = 7.0	***p* = 0.002**	−0.89	[−1.56, −0.23]	r = 0.867
NPRS	30-Day → 90-Day	Wilcoxon	W = 61.5	*p* = 0.734	—	—	—
NPRS	Initial → 90-Day	Wilcoxon	W = 36.0	*p* = 0.096	—	—	—
NPRS	Initial → Endpoint	Wilcoxon	W = 9.5	***p* = 0.002**	−2.56	[−3.83, −1.28]	r = 0.860
Pain	Initial → 30-Day	Wilcoxon	W = 3.5	***p* = 0.002**	−2.90	[−5.10, −0.70]	r = 0.933
Pain	30-Day → 90-Day	Wilcoxon	W = 45.5	*p* = 0.406	—	—	—
Pain	Initial → 90-Day	Wilcoxon	W = 13.0	***p* = 0.023**	−2.20	[−4.56, +0.16]	r = 0.714
Pain	Initial → Endpoint	Wilcoxon	W = 6.5	***p* = 0.001**	−3.75	[−6.31, −1.19]	r = 0.904
Stiffness	Initial → 30-Day	Wilcoxon	W = 0.0	***p* = 0.001**	−1.15	[−1.72, −0.58]	r = 1.000
Stiffness	30-Day → 90-Day	Wilcoxon	W = 44.0	*p* = 0.916	—	—	—
Stiffness	Initial → 90-Day	Paired *t*-test	t = 3.324	***p* = 0.004**	−1.25	[−2.04, −0.46]	d = −0.74
Stiffness	Initial → Endpoint	Wilcoxon	W = 22.0	***p* = 0.003**	−1.45	[−2.99, +0.09]	r = 0.768
Physical Function	Initial → 30-Day	Wilcoxon	W = 7.5	***p* = 0.001**	−5.60	[−8.53, −2.67]	r = 0.902
Physical Function	30-Day → 90-Day	Wilcoxon	W = 84.5	*p* = 0.442	—	—	—
Physical Function	Initial → 90-Day	Wilcoxon	W = 20.5	***p* = 0.008**	−5.90	[−10.44, −1.36]	r = 0.732
Physical Function	Initial → Endpoint	Wilcoxon	W = 5.5	***p* = <0.001**	−11.20	[−16.69, −5.71]	r = 0.936
WOMAC Total	Initial → 30-Day	Wilcoxon	W = 7.0	***p* = <0.001**	−9.65	[−13.97, −5.33]	r = 0.918
WOMAC Total	30-Day → 90-Day	Wilcoxon	W = 84.5	*p* = 0.672	—	—	—
WOMAC Total	Initial → 90-Day	Wilcoxon	W = 15.5	***p* = 0.004**	−9.35	[−15.83, −2.87]	r = 0.797
WOMAC Total	Initial → Endpoint	Wilcoxon	W = 14.0	***p* = <0.001**	−16.40	[−24.48, −8.32]	r = 0.867
QOLS	Initial → 30-Day	Paired *t*-test	t = 0.548	*p* = 0.590	—	—	—
QOLS	30-Day → 90-Day	Paired *t*-test	t = −0.500	*p* = 0.623	—	—	—
QOLS	Initial → 90-Day	Paired *t*-test	t = −0.129	*p* = 0.899	—	—	—
QOLS	Initial → Endpoint	Paired *t*-test	t = −0.563	*p* = 0.580	—	—	—

d = Cohen’s d (paired *t*-test rows only). r = rank-biserial correlation (Wilcoxon rows only), calculated as r = 1 − 4W/n(n + 1); positive values indicate improvement. MD = mean difference (later time point minus earlier). 95% CI from paired t-distribution. — = non-significant or not applicable. Significant *p*—values are bold.
